# Non-invasive Cognitive Enhancement in Epilepsy

**DOI:** 10.3389/fneur.2019.00167

**Published:** 2019-02-27

**Authors:** Claire S. Jacobs, Kim C. Willment, Rani A. Sarkis

**Affiliations:** ^1^Division of Epilepsy, Department of Neurology, Massachusetts General Hospital, Harvard Medical School, Boston, MA, United States; ^2^Department of Neurology, Edward B. Bromfield Epilepsy Program, Brigham and Women's Hospital, Harvard Medical School, Boston, MA, United States

**Keywords:** neuromodulation, stimulants, memantine, donepezil, cognition, memory, epilepsy

## Abstract

Epilepsy patients frequently experience cognitive difficulties, particularly in the domains of memory, attention, and executive function. Despite the frequency of these difficulties among epilepsy patients, current strategies to treat cognitive dysfunction are limited. We performed a systematic review of controlled trials of non-invasive cognitive enhancement in epilepsy. We identified studies examining the efficacy of pharmacological agents, namely the acetylcholinesterase inhibitors donepezil and galantamine, the NMDA non-competitive antagonist memantine, and the stimulant methylphenidate, as well as non-invasive non-pharmacological transcranial magnetic stimulation (TMS) and transcranial direct current stimulation (tDCS). We highlight the data currently available and the limitations of the current literature.

## Introduction

Cognitive concerns are among the most common complaints of patients with both new-onset and chronic epilepsy (1). Cognitive difficulties may affect multiple domains, including memory, language, attention, and executive function (2, 3). These deficits are likely multifactorial, with contributions from ongoing seizures and/or interictal epileptiform discharges, anti-epileptic drugs (AEDs), and the underlying etiology. Potential interventions include avoiding polypharmacy, adjusting AEDs to minimize seizures, interictal discharges and side-effects, cognitive rehabilitation, and potentially, pharmacological agents (4). While pharmacologic agents to improve cognitive function have been studied in the context of disorders such as attention deficit hyperactivity disorder (5) and neurodegenerative disorders (6), the efficacy of these agents in patients with epilepsy has received relatively less attention. This mini-review will discuss evidence for pharmacological agents and non-invasive neurostimulation to compensate for cognitive deficits in patients with epilepsy.

## Materials and Methods

A literature database search was performed as described in supplemental material, and this review was registered on PROSPERO (international prospective register of systematic review). Literature hits were screened for eligibility in two phases based on exclusion and inclusion criteria. All studies were initially screened on title and abstract by two reviewers (CJ, RS). Studies with an applicable title but no available abstract were included for the second phase. In the second phase, the full text was screened, again using the exclusion and inclusion criteria. As shown in Supplemental Figure 1, the search resulted in eight full-length articles meeting inclusion and exclusion criteria. A cohen's d size was calculated for each study when feasible. The eight articles are discussed below and summarized in Tables 1, 2.

**Table 1:** Pharmacologic cognitive enhancement in epilepsy.

**Pharmacologic**	**Trial type**	**Subjects completing study**	**Dosing**	**Cognitive measures**	**Other measures**	**Effect**	**Safety Concerns**
Donepezil	Randomized, double-blinded, placebo-controlled cross-over (7)	23	5–10 mg daily	NAART (baseline); Hopkins Verbal Learning Test-R; CAT; Stroop; Symbol Digit Coding; Grooved Pegboard	POMS; QOLIE-31 & QOLIE-89 memory items	No cognitive effect. Decreased vigor on donepezil (POMS). Subjective memory improvement (both arms)	Statistically significant increase in focal, but not GTC, seizures in both arms
Galantamine	Randomized, double-blinded, placebo-controlled (8)	28: Tx = 13, placebo = 15	4 mg qd, 4 mg bid, or 8/4 mg bid	Verbal Selective Reminding Test; 7–24 Spatial Recall Test	None	No statistically significant change. Trend to improved 7/24 performance	1 subject w increased seizure (likely neoplasm recurrence)
Memantine	Randomized, double-blinded, placebo-controlled (9)	55: Tx = 26, placebo = 29	5 mg daily weeks 1–8, 10 mg daily weeks 9–16	Folstein Mini-Mental State Exam, Wechsler Memory Scale	QOLIE-10-P	Statistically significant MMSE score increase by week 16, and in WMS score by week 8, with further increase by Week 16. Significant difference between doses on within-group analysis^*^	No increase in seizure frequency
Methylphenidate	Double-blind, placebo-controlled single-dose 3-period crossover (10)	31 epilepsy subjects	Single dose of 10 mg and 20 mg (separate visits)	SDMT; MCG; CPT3 (d', HRTSD subtests = primary outcome)	None	Statistically significant increase in SDMT (small effect size) and HRTSD (medium effect) in both treatment arms. Trend to improvement (d'), no change in MCG	No increase in seizure frequency
	One-month open-label (11)	28 epilepsy subjects, 14 non-medicated healthy controls	5 mg bid up to goal 20 mg bid	SDMT; MCG; CPT3	QOLIE-89; BDI-II; BAI; AES; AEP; SSC	Statistically significant improvement with large effect size at visits 1 and 5 for attention measures (omissions and commissions) epilepsy subjects compared vs. controls. Significant subjective quality of life improvements in epilepsy patients	No increase in seizure frequency

*AES, Apathy Evaluation Scale; AEP, Liverpool Adverse Events Profile; BAI, Beck Anxiety Inventory; BDI-II, Beck Depression Inventory second edition; CAT, Continuous Attention Test; CPT3, Conners Continuous Performance Test Third Edition; d', ability to discriminate between target/non-target stimuli (CPT 3 subtest); HRTSD, hit reaction time standard deviation (CPT 3 subtest); MCG, Medical College of Georgia Paragraph Memory Test; NAART, North American Adult Reading Test; POMS, Profile of Mood States; QOLIE, Quality of Life in Epilepsy; SSC, Stimulant Side Effect Checklist; SDMT, Single Digit Modalities Test, Tx, treatment arm. Effect size = Cohen's d effect size, where small effect is 0–0.2 standard deviation difference, medium effect size is 0.2–0.5 SD, and large effect size is 0.5 SDs or above*.

* *Unable to calculate Cohen's d effect size from data presented in referenced study*.

**Table 2:** Non-Invasive neuromodulatory cognitive enhancement in epilepsy.

**Non-pharmacologic**	**Trial type**	**Subjects completing study**	**Dosing**	**Cognitive measures**	**Other measures**	**Effect**	**Safety concerns**
Transcranial magnetic stimulation (TMS)	Randomized, double-blinded, sham-controlled, parallel-design clinical trial (12)	Twenty one MCD subjects: TMS arm = 12, sham arm = 9	rTMS: 1 Hz, 1,200 pulses, 70% of maximum stimulator output intensity over MCD	Digit span (forwards and backwards); Simple reaction time; Stroop test,	Social interaction and energy rating via direct questions	Significant improvement in Stroop (14 subjects), no change in digit span or reaction time. Short-term subjective energy level and social interaction improvement at week 2, but not week 4 or 8.	No. Statistically significant decrease in seizure frequency in rTMS arm at 2 weeks, but not 4 or 8 weeks
Transcranial direct current stimulation (tDCS)	Randomized sham-controlled cross-over study (13)	12 TLE subjects	Slow-oscillatory tDCS (ipsilateral fronto-temporal scalp anode)	Rey Auditory Verbal Learning Test (RAVLT); Rey-Osterrieth Complex Figure Test and Complex Figure B	MOCA; BDI; STAI 1 & 2	Statistically significant improvement in declarative memory (improved retention, large effect size) (*p* = 0.022), slight decrease in forgetfulness (*p* = 0.048) (small effect size)^*^.	No increase in seizures within 1 week after study visits
	Double-blinded, sham-controlled, randomized, parallel-group study (14)	33 TLE subjects: tDCS = 21, sham = 12	Fixed dose tDCS (anode over left dorsolateral prefrontal cortex)	WAIS-III subtests (letters and numbers sequencing, Digits Span Test); RAVLT	Baseline EEG; BDI-II; NDDI-E; QOLIE-31	No effect on cognitive measures. Significant decrease in depressive symptoms in tDCS vs. sham (moderate effect on NDDI-E and BDI-II) at 2 weeks, but not beyond.	No change in seizure frequency 2 or 4 weeks after treatment course

*BDI-II, Beck Depression Inventory second edition; MCD, malformation of cortical development; MOCA, Montreal Cognitive Assessment; NDDI-E, Neurological Disorders Depression Inventory for Epilepsy; QOLIE, Quality of Life in Epilepsy; STAY 1 and 2, State Trait Anxiety; WAIS-R, Wechsler Adult Intelligence Test R. Effect size = Cohen's d effect size, where small effect is 0–0.2 standard deviation difference, medium effect size is 0.2–0.5 SD, and large effect size is 0.5 SDs or above*.

* *Unable to calculate Cohen's d effect size from data presented in referenced study*.

## Pharmacologic Agents

### Acetylcholinesterase Inhibitors

#### Donepezil

Two studies have evaluated donepezil as a cognitive enhancer in epilepsy patients. The first was a 3 month, open-label study of 5–10 mg donepezil in 18 non-pregnant adults with epilepsy on a stable AED regimen (15). Although subjects showed improvement in some cognitive domains, this study was limited by the lack of placebo controls. To follow up on these initial promising results, Hamberger et al. performed a randomized, double-blind, placebo-controlled cross-over trial of donepezil (7). Of the 23 subjects who completed the study (16 male), 17 had focal epilepsy, and 11 were on AED monotherapy alone. Subjects were randomized to either donepezil in months 1–3 and placebo in months 4–6, or vice versa. Donepezil was dosed at 5 mg daily for the first 4 weeks and 10 mg daily for the remaining 8 weeks. Neuropsychological testing and mood assessments were performed at baseline, and again at 2 weeks, 3 and 6 months. The Quality of Life in Epilepsy-31 (QOLIE-31) was administered at baseline, months 3 and 6, while seizure frequency and severity were recorded in seizure diaries. No significant change in memory or other cognitive scores was found with donepezil treatment, and test performances did not correlate with mood, subjective memory, seizure frequency, or adverse events. Subgroup analysis found no significant effect for patients with temporal lobe epilepsy (TLE), non-TLE, mono- or poly-therapy. The authors attribute the significant subjective improvement in the memory subscale of the QOLIE-31 scores reported by both donepezil (*p* = 0.014) and placebo (*p* = 0.001) to placebo effect. The authors also note that, without the placebo control arm, data analysis would likely have shown a positive effect on subjective memory, as in the prior open-label study (15). A statistically significant increase in focal seizure frequency was noted for both arms; from 1.7/mo (SD 5.1) at baseline to 2.6 (SD 5.7) in active arm and 2.5 (SD 5.5) in placebo arm. Frequency of generalized tonic clonic seizures (GTCs) did not change.

#### Galantamine

Twenty-eight subjects (27 with focal onset, 1 with generalized onset, 12 on monotherapy, and 15 on polytherapy) completed this randomized, double-blind, placebo controlled clinical trial; subjects recruited in three successive waves were randomized into either the galantamine (16) or placebo (13) arm (8). The galantamine dose was increased for each successive wave: 4 mg daily in wave 1, 4 mg twice daily in wave 2, and 8 mg AM/4 mg PM in wave 3. Neuropsychological testing was done at baseline and week 12 to evaluate verbal and non-verbal memory. Within the study limits, there was no statistically significant effect on memory by either measure, though the authors noted a trend toward better non-verbal memory performance in the treatment arm at baseline and 12 weeks at the doses tested. Concomitant treatment with AEDs thought to affect cognition had no apparent effect on measures. Subgroup analysis by epilepsy type (TLE or not) was not included, and effect of mood was not evaluated.

### N-Methyl-D-Aspartate (NMDA) Non-competitive Antagonist Memantine

Memantine, a non-competitive NMDA receptor antagonist, has attracted interest due the potential to reduce excitotoxicity-mediated memory deficits. Using a randomized, double-blind, placebo-controlled clinical trial design, Marimuthu et al. recruited epilepsy patients on AEDs and with subjective memory complaints (9). Active arm subjects (*n* = 29) received memantine 5 mg daily for the first 8 weeks and 10 mg daily for the next 8 weeks, while placebo arm subjects (*n* = 30) received a daily placebo for the full 16 weeks. Fifty-five of fifty-nine subjects completed the study (26 active and 29 placebo arm); subjects were between 18 and 55 years old, with different seizure types (GTCs = 39, focal onset impaired awareness = 9, absence seizures = 5, and one each with myoclonic and focal onset preserved awareness seizures). Twenty-one subjects were on AED monotherapy, while thirty four were on polytherapy. Cognitive and memory function were assessed at baseline, and at the end of the 4th, 8th, 12th, and 16th weeks: subjective symptoms were assessed at the same timepoints using the QOLIE-10-P. By week 16, subjects in the active arm experienced an increase in MMSE from 18.42 at baseline to 25.35, compared to an increase from 18.31 to 22.62 in the placebo group (*p* < 0.001). Wechsler memory scale (WMS) score showed a statistically significant improvement from 56.35 to 67.92 by the end of week 8 (*p* = 0.002) and to 91.35 (*p* < 0.001) by the end of week 16; for comparison, placebo arm WMS scores increased from 56.52 to 70.14 by the end of week 16. Within-group analysis between the memantine 5 mg and 10 mg daily periods showed a significant improvement in both MMSE and WMS by week 16 as compared to week 8. Particular areas of improvement in active arm subjects were immediate recall (logical memory), delayed recall (memory span) and visual reproduction memory by week 8, and statistically significant increase in associate learning by the end of week 12. Active arm subjects also reported improved subjective memory compared to placebo arm (*p* = 0.017 by week 8, and *p* = < 0.000 by week 16) and overall QOL improvement (*p* = 0.004 by week 8, *p* < 0.000 in weeks 12 and 16). Of note, cognitive and memory assessment was not adjusted for epilepsy type or mood.

### Psychostimulant Methylphenidate

Given potential cognitive benefits and likely low risk of increased seizures, two studies set out to evaluate methylphenidate as a cognitive enhancer in epilepsy (10, 11). The first was a double-blind, placebo-controlled single-dose 3-period crossover study in 31 adult epilepsy patients (24 with focal, 6 with generalized, and 1 with unclassified epilepsy) with cognitive complaints on a stable AED regimen (10). Subjects completed three study visits ~1 week apart; during each visit, they received either placebo, methylphenidate 10 mg or methylphenidate 20 mg (each subject received each of the three treatments once during the study). Following a 1 h wait for medication absorption and peak effect, subjects completed a neurocognitive battery to evaluate processing speed [Symbol Digit Modalities Test (SDMT)], immediate verbal recall [Medical College of Georgia Paragraph Memory Test (MCG)], and attention-related issues [Conners Continuous Performance Test Third Edition (CPT 3)]. After either dose of methylphenidate, subjects enjoyed a statistically significant cognitive benefit, with comparable benefit noted for both doses. Particular improvement was noted in processing speed and consistency in response speed [hit reaction time standard deviation (HRTSD) subtest of CPT 3]. A trend toward improvement was noted in the go/no-go d' subtest of CPT 3. This study did not evaluate mood, or include subgroup analysis based on epilepsy type.

The authors followed up with a second 1 month open-label trial completed by 28 adult subjects recruited from the crossover study (21 with focal, 6 with generalized, and 1 with unclassified epilepsy) (11). During the open-label phase, subjects started at methylphenidate 5 mg twice daily or 10 mg twice daily, and titrated, as tolerated, toward to 20 mg twice daily (goal dose). Fourteen healthy controls completed neurocognitive testing at the same intervals as epilepsy subjects; controls did not receive methylphenidate. Efficacy, tolerability and adverse effects were assessed on a weekly basis. Following the treatment month, subjects, and healthy controls (when appropriate) repeated the same neurocognitive battery used in the previous cross-over study, while mood was evaluated with the Beck Depression Inventory, 2nd edition (BDI-II), Beck Anxiety Inventory (BAI), and Apathy Evaluation Scale (AES). The QOLIE-89 assessed quality of life in epilepsy subjects only. While there was a significant improvement in both epilepsy subjects and controls across multiple cognitive variables, epilepsy subjects enjoyed greater improvement on measures of attention, and this difference was found to be clinically significant on *post-hoc* analysis. Epilepsy subjects also had significant improvement in quality of life. Both groups had improvements on psychiatric measures, but with no significant difference between the treated epilepsy patients and untreated healthy controls. Mood was not controlled for in the analysis of cognitive improvement, and subgroup analysis based on epilepsy type was not included. Interestingly, scores for the stimulant side effects and the adverse events profile decreased by visit 5 from baseline, which the authors suggest may reflect a general subjective sense of improvement due to either placebo effect or improved quality of life.

## Non-invasive Non-pharmacologic Strategies

We will focus on transcranial direct stimulation (tDCS) and transcranial magnetic stimulation (TMS). Findings are summarized in Table 2. Readers interested in a recent review of invasive neurostimulation effects on cognition and mood in epilepsy are referred to Chan et al. (16).

### Transcranial Magnetic Stimulation (TMS)

Evaluation of TMS to address cognitive deficits experienced by patients with epilepsy has been limited. In their randomized, double-blinded, sham-controlled, parallel-design clinical trial evaluating antiepileptic effects of repetitive TMS (rTMS) on refractory epilepsy patients with malformations of cortical development (MCD), Fregni et al. included cognitive changes as a secondary outcome (12). Twenty-one subjects with drug-refractory, non-surgical epilepsy were randomized to receive 1 Hz rTMS treatment (*n* = 12) or sham treatment (*n* = 9), and underwent daily treatment sessions for 5 consecutive days. Two subjects were on monotherapy, and 19 on polytherapy. In the treatment arm, there was a significant reduction in seizure frequency of up to 72% (*p* = 0.003) at week 2; this effect attenuated with time but did persist until the last check-in at week 8 (58% seizure frequency decrease, *p* = 0.001). Analysis of EEG data revealed a statistically significant decrease in epileptiform discharges in active arm subjects at week 2, which largely washed out by week 8. Cognitive assessment included digit span (forwards and backwards), simple reaction time, and the Stroop test at baseline, after treatment, and 8 weeks after treatment. The 14 subjects able to complete the Stroop showed significant improvement in performance at both post-treatment time-points, but no change in either digit span (*n* = 14) or simple reaction time (*n* = 19). Subjects reported short-term increase in energy and social interaction (subjective measures of cognition) at week 2, but not at weeks 4 or 8; analysis of cognitive improvement was not controlled for changes in energy or social interaction.

### Transcranial Direct Current Stimulation (tDCS)

Transcranial Direct Current Stimulation applies a weak electrical current to the scalp, and thus modifies neuronal membrane potentials in the underlying brain tissue, either by depolarization (anodal tDCS) or hyperpolarization (cathodal tDCS) of the resting potential. **Two** studies have assessed the effect of tDCS on memory in patients with epilepsy. Del Felice et al. performed a randomized sham-controlled cross-over study in 12 subjects with TLE and mesial temporal lobe sclerosis, in which partially sleep-deprived subjects were treated with slow-oscillatory tDCS (sotDCS) prior to napping to evaluate whether sotDCS affects sleep spindles and enhances memory consolidation (13). Subjects underwent neuropsychological assessment and the Montreal Cognitive Assessment (MOCA) prior to active or sham treatment, followed by a period of rest (60 min of sleep or 105 min in bed, whichever occurred first). Active treatment consisted of excitatory depolarizing slow-oscillating anodal stimulation (fluctuating current between 0 and 250 mA, 0.75 Hz frequency applied for 5 min with 1 min interblock interval, 30 min total) over the ipsilateral fronto-temporal region. Thirty minutes after awakening, verbal [Rey Auditory Verbal Learning Test (RAVLT)] and visuospatial recall (Rey-Osterrieth Complex Figure Test and Rey-Osterrieth Complex Figure B) were tested. Subjects self-administered the Beck Depression Inventory (BDI) and State Trait Anxiety Inventory (STAI 1 and 2) between the learning and recall memory testing. Subjects underwent two courses of treatment (one active and one sham), separated by at least 1 week. Investigators found changes to EEG characteristics of certain sleep spindles and increased total sleep time following active stimulation, as well as statistically significant differences on neuropsychiatric testing between the sham and active treatment: declarative memory improved following tDCS and napping, specifically with improved retention (*p* = 0.022), and there was a slight decrease in forgetfulness on visuospatial testing (*p* = 0.048). There were no significant changes in BDI, STAI 1 or STAI 2 scores.

In a 2016 double-blinded, sham-controlled, randomized, parallel-group study, Liu et al. (14) evaluated the efficacy of a 5 day tDCS course on depression and memory in 33 patients with well-controlled TLE. Subjects underwent fixed-dose 2 mA tDCS or sham treatment for 20 min every day for 5 days; the anode was placed over the left dorsolateral prefrontal cortex with the goal of increasing regional activity. Neuropsychological testing [Letters and Numbers Sequencing and Digits Span Test subtests of the Wechsler Adult Intelligent Scale-III (WAIS-III)], symptom inventories [BDI-II, Neurological Disorders Depression Inventory for Epilepsy (NDDI-E)] and a 20 min EEG were performed at baseline, prior to the first session of stimulation, after the last stimulation session, and at the 2 and 4 weeks post-stimulation follow-up sessions. The authors found no difference in the scores of any of the measures of working or verbal memory in active vs. sham arm subjects. Treatment arm subjects reported a statistically significant decrease in depressive symptoms compared to the sham arm subjects, though this difference disappeared by week 2. Interestingly, QOLIE-31 scores did not change between the active and sham arms. The study found no difference in seizure frequency, interictal discharge frequency, or the EEG spectral power in the active arm compared to baseline or the sham treatment arm.

## Conclusions

The memory complaints and broader cognitive difficulties frequently reported by epilepsy patients are multifactorial. Clinicians can support cognitive health in epilepsy patients through judicious selection of AEDs with more benign cognitive side effect profiles (17), minimizing polypharmacy when possible, ensuring appropriate treatment of comorbid mood and sleep disorders, and by actively evaluating patients for potential epilepsy surgery (4, 18).

Despite the prevalence of cognitive complaints among epilepsy patients, there is a relative paucity of studies directly assessing the efficacy of pharmacological or neuromodulatory interventions on cognition. Those studies that have been performed, while a valuable first step, are limited by factors such as limited sample size, heterogeneous patient populations, and different AED regimens and loads. Cognitive performance was not the primary outcome for all studies discussed here, and the cognitive and memory assessment tools varied between the studies, making it difficult to compare interventions directly. In addition, the types of epilepsy varied between studies, making assessment of clinical applicability of these findings difficult as patients with focal vs. generalized epilepsy have distinct cognitive profiles (19, 20) and might differentially benefit from specific agents. Confounding factors such as the effect of mood on cognition were also not controlled for in all but one study. It also has to be kept in mind that patients with subjective cognitive complaints do not always have evidence of objective impairment on formal testing (21). This can impact the patient population participating in trials if it is used as the sole enrollment criterion. Finally, AED polytherapy is also closely linked with fatigue and executive dysfunction; as a result, patients on polytherapy might benefit from a different cognitive enhancement strategy.

The lack of efficacy of acetylcholinesterase inhibitors on cognitive performance in epilepsy is likely due to memory dysfunction in epilepsy being mediated via mechanisms other than loss of cholinergic neurons (7). While stimulants improved mood and processing speed without apparent increase in seizure frequency during the open label time-period of the study, the risks of increased seizure frequency with long-term use and of abuse are unknown and require further study. The encouraging results from the memantine trial also must factor in a clear practice effect for both groups and will need to be replicated.

Non-invasive neuromodulation in epilepsy is appealing due to the potential to impact seizure frequency and/or interictal epileptiform discharge frequency. Of the studies discussed here, although a statistically significant increase in Stroop scores was noted in the active arm of the TMS study in a cohort of patients with MCD, no effects were seen on other cognitive measures. A statistically significant improvement in declarative memory and forgetfulness in TLE patients with mesial temporal sclerosis (MTS) was seen following slow-oscillatory tDCS followed by a nap; while this is encouraging, it is unclear how easily this could be implemented on a chronic basis, or whether it is applicable to patients with other pathologies. Further, in patients with well-controlled TLE, a well-designed tDCS study without a nap did not show clear benefit. Research into non-invasive neuromodulation is ongoing and it may still have a role in cognitive enhancement in epilepsy through reduction in frequency of epileptiform discharges, sleep-dependent memory consolidation, or other mechanisms (22). As with studies of pharmacological intervention, understanding the potential benefit of non-invasive neuromodulation will require larger, appropriately powered, well-designed and controlled studies.

## Author Contributions

CJ and RS contributed to the literature review and examination. CJ wrote the first draft of the manuscript. KW and RS contributed to the conception and design of the work. RS to drafting sections of the manuscript and KW to revising it for important intellectual content. All authors contributed to manuscript revision, read and approved the submitted version.

### Conflict of Interest Statement

The authors declare that the research was conducted in the absence of any commercial or financial relationships that could be construed as a potential conflict of interest.

## References

[1] Thompson PJ Corcoran R. Everyday memory failures in people with epilepsy. Epilepsia (1992) 33(Suppl 6):S18–20. 1486831

[2] Hermann B Seidenberg M Jones J. The neurobehavioural comorbidities of epilepsy: can a natural history be developed? Lancet Neurol. (2008) 7:151–60. 10.1016/S1474-4422(08)70018-8 18207113

[3] Baker GA Taylor J Aldenkamp AP SANAD group. Newly diagnosed epilepsy: cognitive outcome after 12 months. Epilepsia (2011) 52:1084–91. 10.1111/j.1528-1167.2011.03043.x 21453356

[4] Leeman-Markowski BA Schachter SC. Treatment of cognitive deficits in epilepsy. Neurol Clin. (2016) 34:183–204. 10.1016/j.ncl.2015.08.008 26613999

[5] Cortese S Adamo N Del Giovane C Mohr-Jensen C Hayes AJ Carucci S. Comparative efficacy and tolerability of medications for attention-deficit hyperactivity disorder in children, adolescents, and adults: a systematic review and network meta-analysis. Lancet Psychiatry (2018) 5:727–38. 10.1016/S2215-0366(18)30269-4 30097390 PMC6109107

[6] Buckley JS Salpeter SR. A risk-benefit assessment of dementia medications: systematic review of the evidence. Drugs Aging (2015) 32:453–67. 10.1007/s40266-015-0266-9 25941104

[7] Hamberger MJ Palmese CA Scarmeas N Weintraub D Choi H Hirsch LJ. A randomized, double-blind, placebo-controlled trial of donepezil to improve memory in epilepsy. Epilepsia (2007) 48:1283–91. 10.1111/j.1528-1167.2007.01114.x 17484756

[8] Griffith HR Martin R Andrews S LeBron Paige A Ware J Faught E. The safety and tolerability of galantamine in patients with epilepsy and memory difficulties. Epilepsy Behav. (2008) 13:376–80. 10.1016/j.yebeh.2008.05.006 18556248

[9] Marimuthu P Varadarajan S Krishnan M Shanmugam S Kunjuraman G Ravinder J. Evaluating the efficacy of memantine on improving cognitive functions in epileptic patients receiving anti-epileptic drugs: a double-blind placebo-controlled clinical trial (Phase IIIb pilot study). Ann Indian Acad Neurol. (2016) 19:344. 10.4103/0972-2327.179971 27570386 PMC4980957

[10] Adams J Alipio-Jocson V Inoyama K Bartlett V Sandhu S Oso J. Methylphenidate, cognition, and epilepsy: a double-blind, placebo-controlled, single-dose study. Neurology (2017) 88:470–6. 10.1212/WNL.0000000000003564 28031390 PMC5278946

[11] Adams J Alipio-Jocson V Inoyama K Bartlett V Sandhu S Oso J. Methylphenidate, cognition, and epilepsy: a 1-month open-label trial. Epilepsia (2017) 58:2124–32. 10.1111/epi.13917 28990169

[12] Fregni F Otachi PTM Do Valle A Boggio PS Thut G Rigonatti SP. A randomized clinical trial of repetitive transcranial magnetic stimulation in patients with refractory epilepsy. Ann Neurol. (2006) 60:447–55. 10.1002/ana.20950 17068786

[13] Del Felice A Magalini A Masiero S. Slow-oscillatory transcranial direct current stimulation modulates memory in temporal lobe epilepsy by altering sleep spindle generators: a possible rehabilitation tool. Brain Stimul. (2015) 8:567–73. 10.1016/j.brs.2015.01.410 25862600

[14] Liu A Bryant A Jefferson A Friedman D Minhas P Barnard S. Exploring the efficacy of a 5-day course of transcranial direct current stimulation (TDCS) on depression and memory function in patients with well-controlled temporal lobe epilepsy. Epilepsy Behav. (2016) 55:11–20. 10.1016/j.yebeh.2015.10.032 26720704

[15] Fisher RS Bortz JJ Blum DE Duncan B Burke H. A Pilot Study of Donepezil for memory problems in epilepsy. Epilepsy Behav. (2001) 2:330–4. 10.1006/ebeh.2001.0221 12609209

[16] Chan AY Rolston JD Rao VR Chang EF. Effect of neurostimulation on cognition and mood in refractory epilepsy. Epilepsia Open (2018) 3:18–29. 10.1002/epi4.12100 29588984 PMC5839311

[17] Sarkis RA Goksen Y Mu Y Rosner B Lee JW Cognitive and fatigue side effects of anti-epileptic drugs: an analysis of phase III add-on trials. J Neurol. (2018) 265:2137–42. 10.1007/s00415-018-8971-z 30003357

[18] Witt J-A Helmstaedter C. How can we overcome neuropsychological adverse effects of antiepileptic drugs? Expert Opin Pharmacother. (2017) 18:551–4. 10.1080/14656566.2017.1309025 28303728

[19] Sarkis RA Pietras AC Cheung A Baslet G Dworetzky B. Neuropsychological and psychiatric outcomes in poorly controlled idiopathic generalized epilepsy. Epilepsy Behav. (2013) 28:370–3. 10.1016/j.yebeh.2013.05.020 23832134

[20] Loughman A Bowden SC D'Souza W. Cognitive functioning in idiopathic generalised epilepsies: a systematic review and meta-analysis. Neurosci Biobehav Rev. (2014) 43:20–34. 10.1016/j.neubiorev.2014.02.012 24631851

[21] Samarasekera SR Helmstaedter C Reuber M. Cognitive impairment in adults with epilepsy: the relationship between subjective and objective assessments of cognition. Epilepsy Behav. (2015) 52:9–13. 10.1016/j.yebeh.2015.08.013 26398591

[22] Ladenbauer J Ladenbauer J Külzow N de Boor R Avramova E Grittner U. Promoting sleep oscillations and their functionalcoupling by transcranial stimulation enhances memory consolidation in mild cognitive impairment. J Neurosci. (2017) 37:7111–24. 10.1523/JNEUROSCI.0260-17.2017 28637840 PMC6705731

