# Supplementary material for: Non-invasive Cognitive Enhancement in Epilepsy
Source: Front Neurol. 2019 Feb 27;10:167. doi: 10.3389/fneur.2019.00167 (PMC6413707; doi:10.3389/fneur.2019.00167)
Supplement: Supplementary file 1 [file Data_Sheet_1.docx]

Supplementary Material

Mini Review: Noninvasive Cognitive Enhancement in Epilepsy

Claire S. Jacobs, MD PhD, Kim C. Willment, PhD, Rani A. Sarkis*, MD MSc

*** Correspondence:** Rani A. Sarkis, MD MSc; rsarkis@bwh.harvard.edu

# Supplementary Data

The following databases were searched through 4/26/2018: PubMed, EMBASE, Cochrane Central Register of Controlled Trials (CENTRAL), Web of Science, PsycINFO, and ClinicalTrials.gov. This review was registered on the international prospective register of systematic reviews PROSPERO. Databases were searched for published material on randomized controlled trials, controlled clinical trials, or placebo-controlled trials evaluating the effect on cognition in patients with epilepsy of any of the following: modafinil, methylphenidate, dexmethylphenidate, L-lysine-dextroamphetamine, amphetamine, dextroamphetamine, atomoxetine, cholinergic agents, donepezil, rivastigmine, physostigmine, galantamine, and non-invasive neuromodulation. For inclusion, publications had to include objective evaluation of cognitive function in adult patients with epilepsy, and had to be written in English. Studies evaluating only safety of the interventions in epilepsy or their use in disorders other than epilepsy were excluded. The search yielded 5,361 hits (1038 from Pubmed, 2135 from Embase, 1782 from Web of Science, 406 from Cochrane, and none from PsycINFO or ClinicalTrials.gov); 3,288 remained after removing duplicates. Search parameter details are below. The process is summarized in Supplemental Figure 1, a Preferred Reporting Items for Systematic Reviews and Meta-Analyses (PRISMA) flow chart of the literature search.


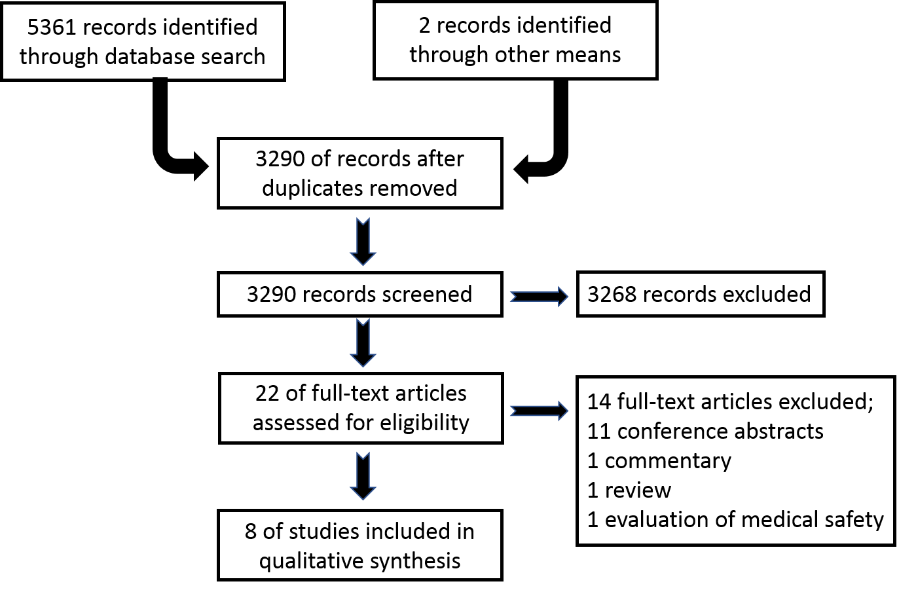


**Supplemental Figure 1:** PRISMA flow chart showing literature search process, including number of records identified via database search and other means, and identification of the included studies.

**PubMed (NCBI)**

20180426

1038 Records

("Epilepsy"[Mesh] OR epilep*[tiab] OR seizur*[tiab])

AND

(

("modafinil"[Supplementary Concept] OR "armodafinil"[Supplementary Concept] OR "Methylphenidate"[Mesh] OR "Dexmethylphenidate Hydrochloride"[Mesh] OR "Dextroamphetamine"[Mesh] OR "Atomoxetine Hydrochloride"[Mesh] OR "donepezil"[Supplementary Concept] OR "Rivastigmine"[Mesh] OR "Physostigmine"[Mesh] OR "Galantamine"[Mesh] OR modafinil[tiab] OR CRL 40476[tiab] OR benzhydrylsulfinylacetamide[tiab] OR provigil[tiab] OR alertec[tiab] OR nuvigil[tiab] OR armodafinil[tiab] OR methylphenidate[tiab] OR ritalin[tiab] OR cotempla[tiab] OR daytrana[tiab] OR biphentin[tiab] OR quillivant[tiab] OR methylin[tiab] OR metadate[tiab] OR aptensio[tiab] OR dexmethylphenidate[tiab] OR focalin[tiab] OR dextroamphetamine[tiab] OR dexedrine[tiab] OR dexamphetamine[tiab] OR dexamfetamine[tiab] OR amphetamine[tiab] OR adderall[tiab] OR evekeo[tiab] OR atomoxetine[tiab] OR tomoxetine[tiab] OR strattera[tiab] OR LY139603[tiab] OR LY 139603[tiab] OR cholinergic[tiab] OR donepezil[tiab] OR aricept[tiab] OR E-2020[tiab] OR rivastigmine[tiab] OR excelon[tiab] OR ENA-713[tiab] OR SDZ-ENA-713[tiab] OR ENA713[tiab] OR physostigmine[tiab] OR eserine[tiab] OR galantamine[tiab] OR razadyne[tiab] OR reminyl[tiab])

OR

("Electric Stimulation Therapy"[mesh:noexp] OR "Transcranial Direct Current Stimulation"[mesh] OR "Transcutaneous Electric Nerve Stimulation"[mesh] OR "Vagus Nerve Stimulation"[mesh] OR direct current stimulat*[tiab] OR tdcs[tiab] OR alternating current stimulat*[tiab] OR transcranial stimulat*[tiab] OR magnetic stimulat*[tiab] OR neuromodulat*[tiab] OR neurostimulat*[tiab] OR electrostimulat*[tiab] OR electrical stimulat*[tiab] OR electric stimulat*[tiab] OR nerve stimulat*[tiab] OR vagus stimulat*[tiab] OR brain stimulat*[tiab] OR cortical stimulat*[tiab] OR trigeminal stimulat*[tiab] OR centromedian thalamic nucleus stimulat*[tiab])

)

AND

("randomized controlled trial"[pt] OR "controlled clinical trial"[pt] OR random*[tiab] OR placebo[tiab] OR trial[tiab] OR groups[tiab])

**Embase (Elsevier; 1974 -)**

20180426

2135 Records

('epilepsy'/exp OR epilep*:ab,ti OR seizur*:ab,ti)

AND

(

('modafinil'/exp OR 'armodafinil'/exp OR 'methylphenidate'/exp OR 'dexmethylphenidate'/exp OR 'dexamphetamine'/exp OR 'atomoxetine'/exp OR 'donepezil'/exp OR 'rivastigmine'/exp OR 'physostigmine'/exp OR 'galantamine'/exp OR modafinil:ab,ti OR 'CRL 40476':ab,ti OR benzhydrylsulfinylacetamide:ab,ti OR provigil:ab,ti OR alertec:ab,ti OR nuvigil:ab,ti OR armodafinil:ab,ti OR methylphenidate:ab,ti OR ritalin:ab,ti OR cotempla:ab,ti OR daytrana:ab,ti OR biphentin:ab,ti OR quillivant:ab,ti OR methylin:ab,ti OR metadate:ab,ti OR aptensio:ab,ti OR dexmethylphenidate:ab,ti OR focalin:ab,ti OR dextroamphetamine:ab,ti OR dexedrine:ab,ti OR dexamphetamine:ab,ti OR dexamfetamine:ab,ti OR amphetamine:ab,ti OR adderall:ab,ti OR evekeo:ab,ti OR atomoxetine:ab,ti OR tomoxetine:ab,ti OR strattera:ab,ti OR LY139603:ab,ti OR 'LY 139603':ab,ti OR cholinergic:ab,ti OR donepezil:ab,ti OR aricept:ab,ti OR 'E-2020':ab,ti OR rivastigmine:ab,ti OR excelon:ab,ti OR 'ENA-713':ab,ti OR 'SDZ-ENA-713':ab,ti OR ENA713:ab,ti OR physostigmine:ab,ti OR eserine:ab,ti OR galantamine:ab,ti OR razadyne:ab,ti OR reminyl:ab,ti OR 'CEP-1538':ab,ti OR 'NSC 73713':ab,ti OR 'LY-135252':ab,ti OR 'LY-139602':ab,ti OR 'ER-4111':ab,ti OR 'SDZ-212-713':ab,ti)

OR

('electrotherapy'/de OR 'nerve stimulation'/de OR 'brain depth stimulation'/exp OR 'functional electrical stimulation'/exp OR 'magnetic stimulation'/exp OR 'nerve cell stimulation'/exp OR 'transcranial direct current stimulation'/exp OR 'transcranial magnetic stimulation'/exp OR 'transcutaneous electrical nerve stimulation'/exp OR 'vagus nerve stimulation'/exp OR tdcs:ab,ti OR ((transcranial OR 'direct current' OR 'alternating current' OR transcranial OR magnetic OR electrical OR electric OR nerve OR vagus OR brain OR cortical OR trigeminal OR 'centromedian thalamic nucleus') NEAR/1 stimulat*):ab,ti OR neuromodulat*:ti,ab OR neurostimulat*:ab,ti OR electrostimulat*:ab,ti)

(

AND

('controlled clinical trial'/exp OR random*:ab,ti OR placebo:ab,ti OR trial:ab,ti OR groups:ab,ti)

**Web of Science (Clarivate Analytics)**

20180426

1782 Records

TS=("epilep*" OR "seizur*")

AND

TS=(

("modafinil" OR "CRL 40476" OR "benzhydrylsulfinylacetamide" OR "provigil" OR "alertec" OR "nuvigil" OR "armodafinil" OR "methylphenidate" OR "ritalin" OR "cotempla" OR "daytrana" OR "biphentin" OR "quillivant" OR "methylin" OR "metadate" OR "aptensio" OR "dexmethylphenidate" OR "focalin" OR "dextroamphetamine" OR "dexedrine" OR "dexamphetamine" OR "dexamfetamine" OR "amphetamine" OR "adderall" OR "evekeo" OR "atomoxetine" OR "tomoxetine" OR "strattera" OR "LY139603" OR "LY 139603" OR "cholinergic" OR "donepezil" OR "aricept" OR "E-2020" OR "rivastigmine" OR "excelon" OR "ENA-713" OR "SDZ-ENA-713" OR "ENA713" OR "physostigmine" OR "eserine" OR "galantamine" OR "razadyne" OR "reminyl" OR "CEP-1538" OR "NSC 73713" OR "LY-135252" OR "LY-139602" OR "ER-4111" OR "SDZ-212-713")

OR

("tdcs" OR (("transcranial" OR "direct current" OR "alternating current" OR "transcranial" OR "magnetic" OR "electrical" OR "electric" OR "nerve" OR "vagus" OR "brain" OR "cortical" OR "trigeminal" OR "centromedian thalamic nucleus") NEAR/1 "stimulat*") OR "neuromodulat*" OR "neurostimulat*" OR "electrostimulat*")

(

AND

TS=("random*" OR "placebo" OR "trial" OR "groups")

**Cochrane Central Register of Controlled Clinical Trials (Wiley)**

20180426

406 Records

("epilep*" OR "seizur*")

AND

("modafinil" OR "CRL 40476" OR "benzhydrylsulfinylacetamide" OR "provigil" OR "alertec" OR "nuvigil" OR "armodafinil" OR "methylphenidate" OR "ritalin" OR "cotempla" OR "daytrana" OR "biphentin" OR "quillivant" OR "methylin" OR "metadate" OR "aptensio" OR "dexmethylphenidate" OR "focalin" OR "dextroamphetamine" OR "dexedrine" OR "dexamphetamine" OR "dexamfetamine" OR "amphetamine" OR "adderall" OR "evekeo" OR "atomoxetine" OR "tomoxetine" OR "strattera" OR "LY139603" OR "LY 139603" OR "cholinergic" OR "donepezil" OR "aricept" OR "E-2020" OR "rivastigmine" OR "excelon" OR "ENA-713" OR "SDZ-ENA-713" OR "ENA713" OR "physostigmine" OR "eserine" OR "galantamine" OR "razadyne" OR "reminyl" OR "CEP-1538" OR "NSC 73713" OR "LY-135252" OR "LY-139602" OR "ER-4111" OR "SDZ-212-713" OR "tdcs" OR (("transcranial" OR "direct current" OR "alternating current" OR "transcranial" OR "magnetic" OR "electrical" OR "electric" OR "nerve" OR "vagus" OR "brain" OR "cortical" OR "trigeminal" OR "centromedian thalamic nucleus") NEAR/1 "stimulat*") OR "neuromodulat*" OR "neurostimulat*" OR "electrostimulat*")

**
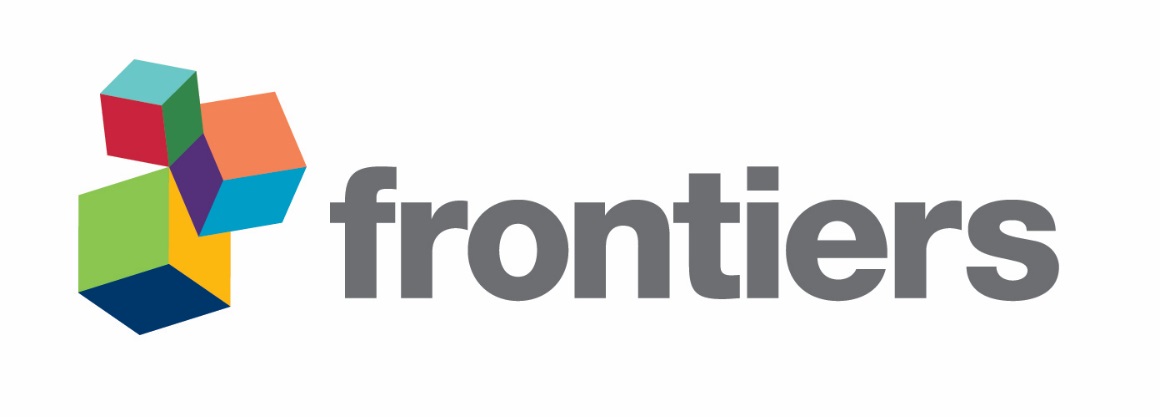
**
